# Incidental recognition of partial anomalous pulmonary venous return involving the left inferior pulmonary vein during central venous catheterization: A case report

**DOI:** 10.1016/j.rmcr.2026.102464

**Published:** 2026-07-09

**Authors:** Lamees AlSulaim, Rasha M. Salama, Bashayer Alzayed, Batool Alshanqiti, Abdulaziz H. Alzeer

**Affiliations:** a Department of Surgery, College of Medicine, Qassim University, Qassim, Saudi Arabia; b Department of Anatomy and Histology, College of Medicine, Qassim University, Qassim, Saudi Arabia; c Department of Critical Care, King Khalid University Hospital, King Saud University Medical City, Riyadh, Saudi Arabia

## Abstract

Partial anomalous pulmonary venous return (PAPVR) is a rare congenital anomaly that may complicate central venous access by predisposing to catheter malposition. We report a 70-year-old woman in whom a left internal jugular dialysis catheter was inadvertently advanced into the left inferior pulmonary vein through an anomalous connection with the left innominate vein. Computed tomography (CT) angiography confirmed PAPVR, and the catheter was safely removed. This case illustrates the procedural risks of unrecognized venous anomalies and emphasizes the importance of CT imaging and vigilance when catheter trajectories deviate from expected anatomical pathways.

## 1 Introduction

Partial anomalous pulmonary venous return (PAPVR) is a rare congenital cardiovascular anomaly in which one or more pulmonary veins, that normally carry oxygenated blood from the lungs to the heart's left atrium, instead follows an anomalous oblique trajectory within the posterior mediastinum and drain abnormally into the systemic venous system or, less commonly, to the right atrium. PAPVR often coexists with atrial septal defects, particularly of the sinus venosus type, and may remain clinically silent [1]. The anomaly has an estimated prevalence of 0.4–0.7% in the general population, with left-sided variants accounting for approximately 10% of cases [2]. Most cases are detected incidentally during imaging performed for unrelated indications. The resultant left-to-right shunt may progressively cause right heart volume overload and, in some instances, lead to pulmonary hypertension [3].

In critically ill patients requiring central venous access, unrecognized PAPVR poses a potential procedural hazard, as catheters introduced through the jugular or subclavian veins may follow aberrant venous pathways, resulting in malposition. Herein, we report a case of PAPVR where a left internal jugular dialysis catheter inadvertently was advanced into the left lower pulmonary vein via an anomalous connection with the left innominate vein, emphasizing the procedural risks associated with unrecognized venous anomalies and the pivotal role of imaging in their identification.

## 2 Case report

A 70-year-old woman was admitted to the intensive care unit (ICU) with community acquired pneumonia that required prolonged mechanical ventilation. Her past medical history was remarkable for type 2 diabetes mellitus, systemic hypertension, and chronic kidney disease. During her ICU course, she developed acute kidney injury necessitating hemodialysis. A left internal jugular vein (IJV) dialysis catheter was inserted in the standard fashion under ultrasound (US) guidance, achieving adequate blood flow.

**Fig. 1:**
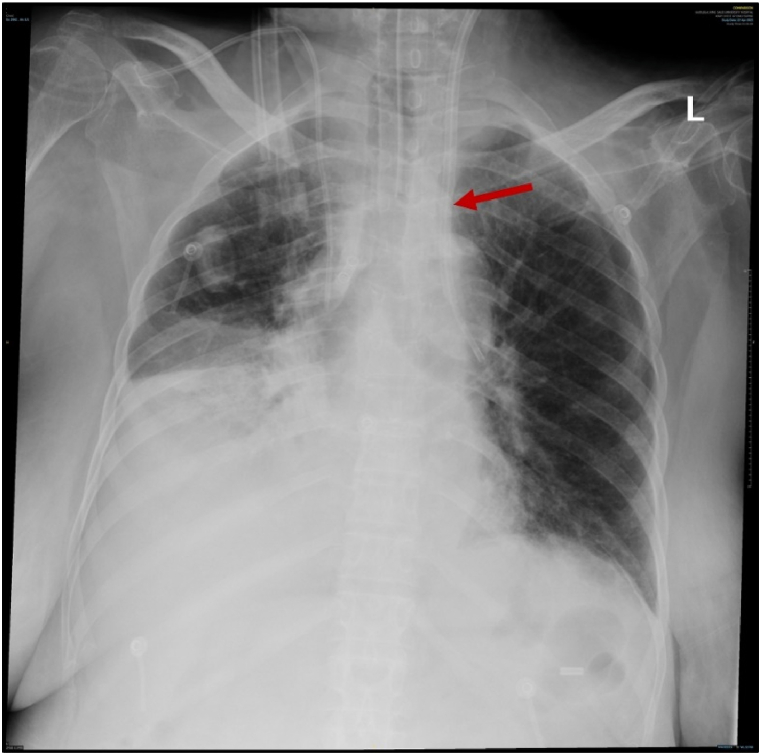
Chest X-ray demonstrating the left IJV central dialysis catheter abnormally located in the left mediastinum as indicated by the red arrow.

**Fig. 2:**
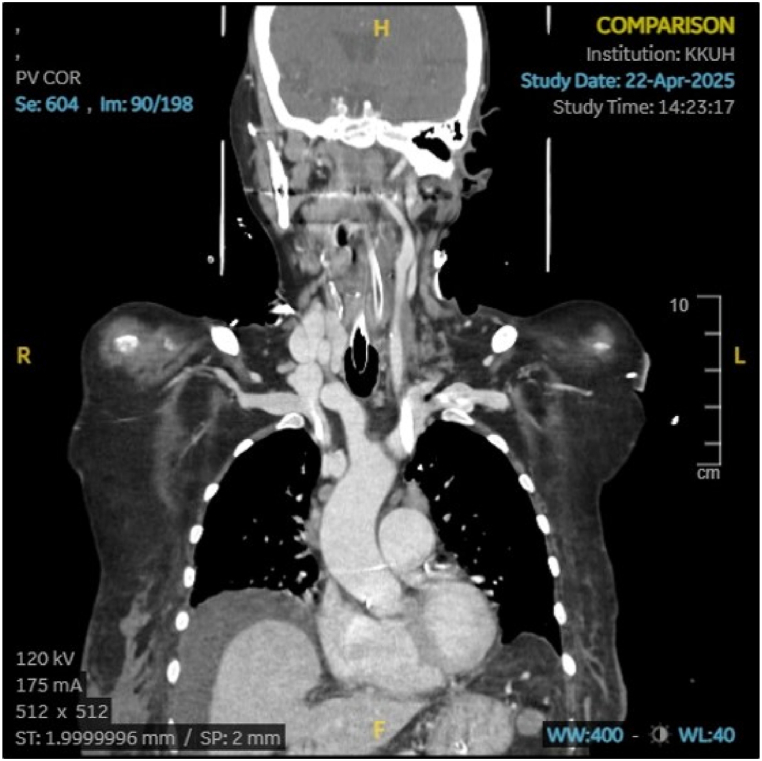
CT angiography coronal image of the chest and neck depict that the dialysis catheter **traversed** the left internal jugular vein into the left innominate vein, then deviated extraluminally through the left mediastinum before terminating within the left lower pulmonary vein.

A chest radiograph obtained to verify catheter positioning demonstrated that the catheter tip failed to cross the midline into the left brachiocephalic vein and instead projected leftward along the contour of the aortic arch, raising concern for malposition (Fig. 1). Bedside ultrasound confirmed correct intraluminal placement within the left IJV. Nevertheless, aspiration yielded non-pulsatile bright red blood, and subsequent venous blood gas analysis demonstrated pH 7.32, PCO_2_ 65 mmHg, and PO_2_ 102 mmHg, findings inconsistent with standard venous values. This led to a suspicion of intra-arterial cannulation or the existence of a left-to-right shunt. A vascular surgeon was consulted, and subsequent computed tomography (CT) angiography of the chest and neck revealed that the catheter traversed from the left IJV into the left innominate vein, deviated extraluminally into the left mediastinum, and terminated within the left lower pulmonary vein (Fig. 2). The proximal catheter port was located within the mediastinum and the distal port within the pulmonary vein. CT findings revealed an anomalous venous connection between the left innominate vein and the left lower pulmonary vein, consistent with PAPVR, which accounted for the aberrant catheter trajectory. Following confirmation that the catheter was not positioned within an arterial structure, it was subsequently removed under sterile conditions without ensuing complications and the patient was dialyzed through a femoral venous catheter.

## 3 Discussion

PAPVR is an uncommon congenital cardiovascular anomaly, most often identified incidentally during imaging or invasive procedures performed for unrelated indications, particularly in patients without other cardiovascular abnormalities [1]. Embryologically, it arises when the left upper pulmonary vein common pulmonary vein fails to fully incorporate into the left atrium, and maintain anomalous drainage into the systemic venous circulation mainly the left innominate vein to ultimately drain into the right atrium allowing some pulmonary veins to. Although they are often clinically silent, this variant carries important procedural implications, particularly in critically ill patients requiring central venous access. Anomalous venous pathways can inadvertently guide catheters introduced via the internal jugular or subclavian veins into pulmonary venous structures, resulting in catheter malposition with potential complications such as ineffective dialysis, vascular injury, pulmonary hemorrhage, or mediastinal bleeding.

In this case, bright blood aspirated from the left IJV catheter demonstrated arterial oxygen saturation, whereas bedside US had confirmed appropriate venous entry. Subsequent CT angiography delineated an anomalous communication between the left innominate and left lower pulmonary veins via a vertical vein, consistent with PAPVR and explaining the advancement of the dialysis catheter into the pulmonary venous system, a rare but clinically significant occurrence.

Left-sided variants of PAPVR most commonly involve drainage of the left upper pulmonary vein into the left brachiocephalic vein through a vertical channel. A large CT series of 45,538 studies reported a PAPVR prevalence of **0.1%,** demonstrating a predominance of left upper lobe involvement (47%), with left lower lobe drainage observed in only a 2%, highlighting the rarity of inferior-vein variants [4]. The anomalous connection observed in our case between the left brachiocephalic vein and the left inferior pulmonary vein represents an exceptionally rare configuration not previously described in published case reports, broadening the recognized spectrum of pulmonary venous drainage anomalies relevant to central venous access and imaging evaluation.

These findings highlight the diagnostic challenge posed by unrecognized venous anomalies during central line placement. In such cases, cross-sectional imaging plays a decisive role in clarifying unexpected catheter trajectories and confirming the underlying anatomical variation. A systematic diagnostic approach supported by CT angiography was pivotal in delineating the aberrant anatomy, confirming the diagnosis, and guiding catheter management [5]. CT remains the reference standard for diagnosing PAPVR, and cardiac magnetic resonance imaging (MRI) offers complementary functional assessment [6]. Although transesophageal echocardiography (TEE) enables dynamic evaluation of intracardiac venous inflow, CT and MRI remain superior for identifying extracardiac or mediastinal variants.

Given the overall scarcity of such findings, recognition of PAPVR during central venous catheterization has remained exceptionally rare since its initial description in 1949, with only a few cases documented in the literature, despite the widespread and routine use of the procedure [5,7]. The expanding use of CT and MRI is expected to increase the detection of PAPVR and incidental catheter malposition, thereby enhancing early recognition of such vascular anomalies [6]. In the absence of standardized management guidelines and given limited safety data, most authors advocate catheter removal once malposition is confirmed [5,8]. Consistent with this approach, the catheter was removed in our patient, and alternative venous access was subsequently recommended for future similar situations. This case adds to the limited body of literature by demonstrating a previously unreported inferior-vein variant of PAPVR and reaffirming the diagnostic value of CT imaging as well as the need for vigilance when catheter trajectories deviate from expected anatomical pathways.

### 3.1 Summary

This case highlights the need to recognize PAPVR as an uncommon yet clinically significant cause of catheter malposition during central venous access. CT angiography provides definitive diagnosis through precise delineation of venous anatomy, while procedural safety depends on heightened clinical vigilance, meticulous US-guided technique, and confirmatory imaging.

## CRediT authorship contribution statement

**Lamees AlSulaim:** Conceptualization, Formal analysis, Writing – original draft, Writing – review & editing. **Rasha M. Salama:** Conceptualization, Writing – original draft. **Bashayer Alzayed:** Writing – original draft. **Batool Alshanqiti:** Conceptualization, Writing – original draft. **Abdulaziz H. Alzeer:** Supervision, Writing – original draft, Writing – review & editing.

## Declaration of competing interest

The authors declare that they have no known competing financial interests or personal relationships that could have appeared to influence the work reported in this paper.
